# Supplementary material for: Inhaling Peppermint Essential Oil as a Promising Complementary Therapy in the Treatment of Nausea and Vomiting
Source: J Clin Med. 2025 Jul 17;14(14):5069. doi: 10.3390/jcm14145069 (PMC12294836; doi:10.3390/jcm14145069)
Supplement: Supplementary file 1 [file jcm-14-05069-s001.zip › jcm-3727223 - Supplementary Materials.pdf]

Supplementary Material

Inhaling Peppermint Essential Oil is Beneficial in the Treatment of Nausea and Vomiting:

A Systematic Review and Meta-analysis of Randomized Clinical Trials

Supplementary Figures: 14, Supplementary Tables: 6

FIGURE LEGENDS

Figure S1 Sensitivity analysis of the severity of nausea and vomiting of postoperative patients, excluding the article of Cetin (MD = mean difference)

Figure S2 Moving averages of peppermint oil intervention versus control for PONV scores over time (hours)

Figure S3 Individual study results comparing peppermint oil intervention versus control for PONV scores over time (hours)

Figure S4 Individual study results comparing peppermint oil intervention versus control for PONV scores over time (minutes)

Figure S5 Moving averages of peppermint oil intervention versus control for NVP scores over time (days)

Figure S6 Individual study results comparing peppermint oil intervention versus control for NVP scores over time (days)

Figure S7 Moving averages of peppermint oil intervention versus control for CINV scores over time (days)

Figure S8 Individual study results comparing peppermint oil intervention versus control for CINV scores over time (days)

Figure S9 Risk of bias assessment of the included postoperative studies using the revised Cochrane risk-of-bias tool (RoB2)

Figure S10 Risk of bias assessment of the included postoperative studies using the revised Cochrane risk-of-bias tool (RoB2) shown in percentage terms

Figure S11 Risk of bias assessment of the included pregnancy studies using the revised Cochrane risk-of-bias tool (RoB2)

Figure S12 Risk of bias assessment of the included pregnancy studies using the revised Cochrane risk-of-bias tool (RoB2) shown in percentage terms

Figure S13 Risk of bias assessment of the included chemotherapy studies using the revised Cochrane risk-of-bias tool (RoB2)

Figure S14 Risk of bias assessment of the included chemotherapy studies using the revised Cochrane risk-of-bias tool (RoB2) shown in percentage terms

TABLE LEGENDS

Table S1 Versions of the search key in different databases

Table S2 Types and original ranges of measurement tools evaluating nausea and vomiting

Table S3 Basic characteristics of the postoperative studies

Table S4 Basic characteristics of the pregnancy studies

Table S5 Basic characteristics of the chemotherapy studies

Table S6 PRISMA 2020 Checklist

Table S1 Versions of the search key in different databases

**Searchkey used:**
**((peppermint) OR (Mentha piperita)) AND ((nausea) OR (vomiting))**
**Pubmed**<br>*Used the advanced search builder without restrictions.*
("mentha piperita"[MeSH Terms] OR ("mentha"[All Fields] AND "piperita"[All Fields]) OR "mentha piperita"[All Fields] OR "peppermint"[All Fields] OR ("mentha piperita"[MeSH Terms] OR ("mentha"[All Fields] AND "piperita"[All Fields]) OR "mentha piperita"[All Fields])) AND ("nausea"[MeSH Terms] OR "nausea"[All Fields] OR "nauseas"[All Fields] OR ("vomiter"[All Fields] OR "vomiters"[All Fields] OR "vomiting"[MeSH Terms] OR "vomiting"[All Fields] OR "vomit"[All Fields] OR "vomited"[All Fields] OR "vomits"[All Fields] OR "vomitings"[All Fields] OR "vomition"[All Fields] OR "vomitting"[All Fields]))
**EMBASE**<br>*Used the advanced search builder with no restrictions and unchecked mapping.*
('peppermint'/exp OR peppermint OR 'mentha piperita'/exp OR 'mentha piperita' OR (('mentha'/exp OR mentha) AND piperita)) AND ('nausea'/exp OR nausea OR 'vomiting'/exp OR vomiting)
**Scopus**<br>*Used the advanced search builder without restrictions.*
((peppermint) OR (mentha AND piperita)) AND ((nausea) OR (vomiting))
**Web of Science**<br>*Used the advanced search builder without restrictions.*
ALL=(((peppermint) OR (Mentha piperita)) AND ((nausea) OR (vomiting)))
**Cochrane Library (TRIALS)**<br>*Used the advanced search within All text without restrictions.*
((peppermint) OR (Mentha piperita)) AND ((nausea) OR (vomiting))

Table S2 Types and original ranges of measurement tools evaluating nausea and vomiting

Study	Nausea and vomiting type	Scale type	Range	Scale description
**Ahmadi et al., 2020 [28]**	postoperative	Visual analog scale for nausea	0-100	Zero is equivalent to the absence of nausea, and one hundred indicates the highest severity of nausea.
**Maghami et al., 2020 [33]**	postoperative	Nausea and vomiting assessing scale	0-100	The nausea severity scores ranged from zero (no nausea) to 100 (most severe nausea)
**Ferruggiari et al., 2012 [55]**	postoperative	Visual analog scale for nausea	0-200	The degree of nausea ranged from „no nausea" at the 0-mm mark to „worst possible nausea" at the 200-mm mark.
**Aydin et al. 2018 [52]**	postoperative	Visual analog scale for nausea	0-100	The patient was asked to mark his/her condition on a 100 mm scale. 0-1: no nausea, 2-4: mild, 5-7: moderate, and 8-10: severe nausea.
**Lane et al. 2012 [57]**	postoperative	6 points descriptive ordinal rating scale	0-5	The scale measured the participants' subjective perceptions of nausea and vomiting symptoms from "I am not experiencing any nausea" to "I vomited."
**Anderson et al. 2004 [51]**	postoperative	Visual analog scale for nausea	0-100	The patients marked their symptoms on a 100-mm long line on paper. The two ends were marked as "no nausea" and "worst possible nausea."
**Baek et al. 2024 [53]**	postoperative	Halpin nausea and vomiting scale	0-5	The scale was developed to assess the severity of vomiting. Each rating corresponds to the frequency and intensity of vomiting episodes over a 12-hour period, ranging from no vomiting (0) to severe intractable vomiting with more than 7 episodes (5).
**Imani et al. 2024 [56]**	postoperative	Likert scale	0-10	Numerical rating scale, the Likert-type scale scored from zero (minimal or no symptom) to four (worst symptom)
**Cetin et al. 2024 [54]**	postoperative	Visual analog scale for nausea	0-10	A scale from 0 (no nausea) to 10 (very severe nausea).
**Sites et al. 2014 [58]**	postoperative	Descriptive ordinal scale	0-10	On the scale, 0 represented the absence of symptoms, and 10 denoted the most severe symptoms.
**Tate et al. 1997 [59]**	postoperative	Nausea score	0-4	The scale is from 0, as I am not experiencing any nausea, to 4, I am so nauseated, I feel I am about to vomit
**Amzajerdi et al., 2022 [29]**	pregnancy	Rhodes nausea and vomiting questionnaire	0-32	Eight 5-point self-report items. Likert-type scale for each item is scored from zero (minimal or no symptom) to four (worst symptom). The item scores are summed for a total score.
**Joulaeerad et al., 2017 [32]**	pregnancy	Pregnancy Unique Quantification of Emesis/Nausea Questionnaire	3-15	Three PUQE questions. Each has a rating from 1–5. A total score between 3–6 points is mild, 7–12 points is moderate, and ≥13 points is severe NVP.
**Pasha et al., 2012 [63]**	pregnancy	Visual analog scale for nausea	0-10	Scores 0 and 10 are respectively indicative of the best and the worst condition.
**Ertürk et al. 2021 [30]**	chemotherapy	Visual analog scale for nausea	0-10	The patient was asked to place a mark on the 10cm scale to indicate the level of intensity of his/her nausea. 0 means no nausea, and 10 means severe nausea.
**Jafarimanesh et al. 2020 [31]**	chemotherapy	Visual analog scale for nausea	0-10	A 10-centimeter line ranged from "0 = no feeling of nausea" to "10 = severe feeling of nausea".
**Eghbali et al. 2017 [60]**	chemotherapy	Rhodes' index	0-4	The patient completed the questionnaire with a score from minimal or no signs (score 0) to the most severe condition (score 4).
**Mapp et al. 2020 [62]**	chemotherapy	Baxter Retching Faces pictorial scale	0-10	The 6-point pictorial scale depicts varying stages of nausea with possible scores from 0 (neutral) to 10 (emesis).
**Lestari et al. 2017 [61]**	chemotherapy	Rodhes of Index Nausea, Vomiting, and Retching (RINVR)	0-32	0= not Nausea and Vomiting; 1-8 = Lightweight; 9-16 = medium; 17-24 = heavy; 25-32 = bad

Table S3 Basic characteristics of the postoperative studies

First Author, Publication year	Country	Sample size (intervention /placebo)	Age (years; mean ± SD)	Sex (female % of total)	Surgical procedure	Drug type	Dosage	Follow up period	Frequency of intervention
**Ahmadi et al. 2020 [28]**	Iran	40/40	46.4 ±12.1	50.8	abdominal	peppermint EO	2 drops (0.1mL) of 10% EO +2cc DW	10 minutes	once for 5 minutes
**Maghami et al. 2020 [33]**	Iran	30/26	I: 62.4 ±10.22 C: 57.54 ±8.94	30.4	open-heart	peppermint EO	0.1mL of EO plus 10mL DW	0-4, 4-8 & 8-12 hours	10 minutes before every examination
**Ferruggiari et al. 2012 [55]**	USA	23/22	ND	100	any	peppermint EO	2 drops (0.1mL) of EO and 5mL of 0.9% normal saline	5 and 10 minutes	once for 5 minutes
**Aydin et al. 2018 [52]**	Turkey	27/29	I: 50,56 ±20,81 C:48,14 ±23,1	48.2	head, neck, eye, ear, and intraabdominal	peppermint EO	EO was diluted to 1/10 with wheat oil	0-2, 2-6, 6-12, 12-24, and 24-48 hours	5 times in every 30 min
**Lane et al. 2012 [57]**	USA	22/8	31,3 (ranged from 22 to 43 years)	100	post C-section	peppermint spirit	1mL peppermint spirit	2 and 5 minutes	at baseline, at 2 minutes and at 5 minutes
**Anderson et al. 2004 [51]**	USA	10/12	I: 42±6 C: 44±5	63.6	any	peppermint EO	0.2mL of EO and 2mL of isotonic saline	2 and 5 minutes	once, taking three slow, deep breaths
**Baek et al., 2025 [53]**	South Korea	30/30	I: 75.4 ± 6.4 C: 73.8 ± 8.4	15	total knee arthroplasty	peppermint EO	5 drops (0.25mL) of 100% pure aroma oil	24, 48, 72 hours	at least five nasal inspirations
**Imani et al. 2024 [56]**	Iran	70/70	I: 51.92±15.62 C: 51.47±13.25	72.14	laparoscopic cholecystectomy	peppermint EO	3 drops (0.15mL) of 100% EO	24, 48 hours	5 minutes repeated 3 times
**Cetin et al. 2024 [54]**	Turkey	38/38	I: 42.11±7.13 C: 44.89±9.57	50	cervical	peppermint EO	5 drops (0.25mL) of EO	5, 35, 65, 95 minutes, 2, 6, 12, 24 hours	replaced every 30 minutes.
**Sites et al. 2014 [58]**	USA	26/16	I: 47.8±15.3 C: 45.7±17.1	88.1	laparoscopic, ear, nose and throat, orthopedic, or urological	peppermint spirit	0.5mL peppermint spirit	5 and 10 minutes	3 repetitions of deep breathing
**Tate et al. 1997 [59]**	UK	6/6/6	I1: 45.5 I2: 43.2 C: 54.0	100	gynaecological	peppermint EO, peppermint essence	not defined	24, 48 hours	inhaled from the bottle when feeling nauseous

EO=essential oil, I=interventional group, C=control group, mL=milliliter, cc=cubic centimeter, DW=distilled water

Table S4 Basic characteristics of the pregnancy studies

First Author, Publication year	Country	Sample size (intervention /placebo)	Age (years; mean ± SD)	Gestational age (weeks, mean ± SD)	Drug type	Dosage	Follow up period	Frequency of intervention
**Amzajerdi et al. 2022 [29]**	Iran	33/33	I: 26.30 ±4.57<br>C: 27.79 ±3.51	I: 10.31 (2.50)<br>C: 10.64 (2.34)	Peppermint EO	4 drops (0.2mL) of EO diluted to 10% in sesame oil	7 days	twice a day
**Joulaeerad et al. 2017 [32]**	Italy	28/28	I: 26.39 ±4.27<br>C: 27.79 ±3.51	I: 12.4±3.77<br>C: 12.1±4.06	Peppermint EO	5 drops (0.25mL) of EO diluted to 10% in sweet almond oil	4 days	4 times a day
**Pasha et al. 2012 [63]**	Iran	30/30	I: 24.8 ±3.56<br>C: 25.1 ±4.76	I: 9.07 ± 1.31<br>C: 9.73 ± 2.21	Peppermint EO	4 drops (0.2mL) of EO in a bowl of water	4 days	whole night under the bed

EO=essential oil, I=interventional group, C=control group, mL=milliliter

Table S5 Basic characteristics of the chemotherapy studies

First Author, Publication year	Country	Sample size (intervention /placebo)	Age (years; mean ± SD)	Sex (female % of total)	Cancer type	Drug type	Dosage	Follow up period	Frequency of intervention
**Mapp et al., 2020 [62]**	USA	37/42	I: 51.0±15.2<br>C: 54.1±13.8	72.16	any, except: head and neck	peppermint EO	cool damp washcloth with two drops (0.1mL) of EO	30 minutes	once
**Lestari et al., 2017 [61]**	Indonesia	150/135	I: 21-70<br>C: 31-70	81.3	liver, cervical, lung, nasopharyngeal, breast, colon, melanoma, lymphoma, sarcoma,	peppermint EO	EO dropped onto a cotton ball	5 minutes	once
**Ertürk et al. 2021 [30]**	Turkey	36/44	I: 49,94 ±10,47<br>C: 54,63 ±10,15	67.5	any	peppermint EO, sweet almond oil	1 drop (0.05mL) of aromatic mixture	5 days	3 times a day
**Jafarimanesh et al. 2020 [31]**	Iran	42/42	I: 49,6 ±11,78<br>C: 51,9 ±9,52	100	breast	peppermint extract in tap water	40 drops (2mL) of peppermint extract in 20cc of tap water	1 and 2 days	every 8 hours
**Eghbali et al. 2017 [60]**	Iran	50/50	47,86±9,52	100	breast	Peppermint EO	2 drops (0.1mL) of EO	1-5 days	20 minutes three times a day.

EO=essential oil, I=interventional group, C=control group, mL=milliliter, cc=cubic centimeter

Study	Comment	Peppermint oil N	Peppermint oil Mean	Peppermint oil SD	Control N	Control Mean	Control SD	MD	95% CI	Weight
**Baseline**										
Baek et al., 2024_Baseline	numerical scale	30	4.40	4.40	30	6.40	4.60	-2.00	[-4.28; 0.28]	10.93%
Imani et al., 2024_Baseline	numerical scale	70	4.37	1.64	70	4.30	1.75	0.07	[-0.49; 0.63]	20.47%
Ferruggiari et al., 2012_Baseline	VAS	23	6.25	2.96	22	5.95	3.06	0.30	[-1.46; 2.06]	13.66%
Anderson et al., 2004_Baseline	VAS	10	6.25	1.05	12	5.79	0.58	0.47	[-0.26; 1.19]	19.71%
Lane et al., 2012_Baseline	numerical scale	22	6.72	1.66	8	6.25	1.86	0.47	[-0.99; 1.93]	15.42%
Ahmadi et al., 2020_Baseline	VAS	40	6.03	1.50	40	5.07	1.74	0.96	[0.25; 1.67]	19.80%
**Random effects model**		**195**			**182**			**0.36**	**[-0.30; 1.01]**	**100.00%**
Heterogeneity: I^2 = 37% [0%; 75%], τ^2 = 0.08, p = 0.158										
Test for effect in subgroup: t_5 = 1.41 (p = 0.218)										
**5 min**										
Lane et al., 2012_5 min	numerical scale	22	1.82	2.56	8	7.00	1.42	-5.18	[-6.63; -3.73]	31.97%
Anderson et al., 2004_5 min	VAS	10	2.94	1.02	12	2.81	0.74	0.13	[-0.63; 0.89]	40.39%
Ferruggiari et al., 2012_5 min	VAS	23	4.00	2.91	22	3.75	3.26	0.25	[-1.56; 2.06]	27.64%
**Random effects model**		**55**			**42**			**-1.59**	**[-9.29; 6.10]**	**100.00%**
Heterogeneity: I^2 = 95% [89%; 98%], τ^2 = 9.06, p < 0.001										
Test for effect in subgroup: t_2 = -0.89 (p = 0.467)										
**0-2 hours**										
Maghami et al., 2020_0-2 hours	questionnaire	30	2.43	2.84	26	4.61	2.85	-2.18	[-3.67; -0.69]	45.78%
Aydin et al., 2018_0-2 hours	VAS	27	0.67	1.24	29	1.59	2.53	-0.92	[-1.95; 0.11]	54.22%
**Random effects model**		**57**			**55**			**-1.43**	**[-9.29; 6.43]**	**100.00%**
Heterogeneity: I^2 = 46%, τ^2 = 0.36, p = 0.174										
Test for effect in subgroup: t_1 = -2.31 (p = 0.260)										
**2-6 hours**										
Aydin et al., 2018_2-6 hours	VAS	27	0.78	1.50	29	1.45	1.38	-0.67	[-1.43; 0.09]	56.43%
Maghami et al., 2020_2-6 hours	questionnaire	30	2.20	2.83	26	2.68	2.93	-0.48	[-1.99; 1.03]	43.57%
**Random effects model**		**57**			**55**			**-0.63**	**[-1.60; 0.33]**	**100.00%**
Heterogeneity: I^2 = 0%, τ^2 = 0, p = 0.826										
Test for effect in subgroup: t_1 = -8.32 (p = 0.076)										
**6-12 hours**										
Aydin et al., 2018_6-12 hours	VAS	27	0.15	0.46	29	2.24	2.59	-2.09	[-3.05; -1.13]	38.01%
Baek et al., 2024_6-12 hours	numerical scale	30	3.00	3.80	30	4.60	3.60	-1.60	[-3.47; 0.27]	26.78%
Maghami et al., 2020_8-12 hours	questionnaire	30	2.00	2.60	26	1.61	1.92	0.39	[-0.80; 1.58]	35.21%
**Random effects model**		**87**			**85**			**-1.09**	**[-4.44; 2.27]**	**100.00%**
Heterogeneity: I^2 = 81% [40%; 94%], τ^2 = 1.49, p = 0.006										
Test for effect in subgroup: t_2 = -1.39 (p = 0.299)										
**12-24 hours**										
Aydin et al., 2018_12-24 hours	VAS	27	0.07	0.38	29	2.55	3.78	-2.48	[-3.86; -1.10]	30.23%
Imani et al., 2024_12-24 hours	numerical scale	70	1.91	0.97	70	2.70	1.33	-0.79	[-1.18; -0.40]	40.09%
Baek et al., 2024_12-24 hours	numerical scale	30	1.80	2.40	30	2.40	3.20	-0.60	[-2.03; 0.83]	29.67%
**Random effects model**		**127**			**129**			**-1.19**	**[-3.57; 1.18]**	**100.00%**
Heterogeneity: I^2 = 64% [0%; 90%], τ^2 = 0.55, p = 0.064										
Test for effect in subgroup: t_2 = -2.16 (p = 0.164)										
**24-48 hours**										
Aydin et al., 2018_24-48 hours	VAS	27	0.00	0.01	29	0.62	1.08	-0.62	[-1.01; -0.23]	34.22%
Imani et al., 2024_24-48 hours	numerical scale	70	0.70	0.92	70	1.25	1.23	-0.55	[-0.91; -0.19]	34.38%
Baek et al., 2024_24-48 hours	numerical scale	30	0.80	2.00	30	0.40	1.00	0.40	[-0.40; 1.20]	31.40%
**Random effects model**		**127**			**129**			**-0.37**	**[-1.61; 0.88]**	**100.00%**
Heterogeneity: I^2 = 62% [0%; 89%], τ^2 = 0.14, p = 0.071										
Test for effect in subgroup: t_2 = -1.26 (p = 0.335)										

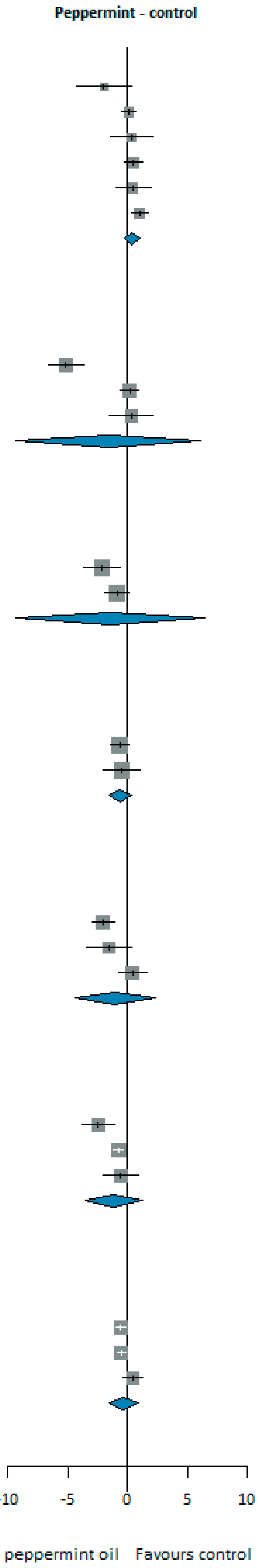

Figure S1 Sensitivity analysis of the severity of nausea and vomiting of postoperative patients, excluding the article of Cetin (MD = mean difference, CI = confidence interval) [28,33,51–53,55–57]

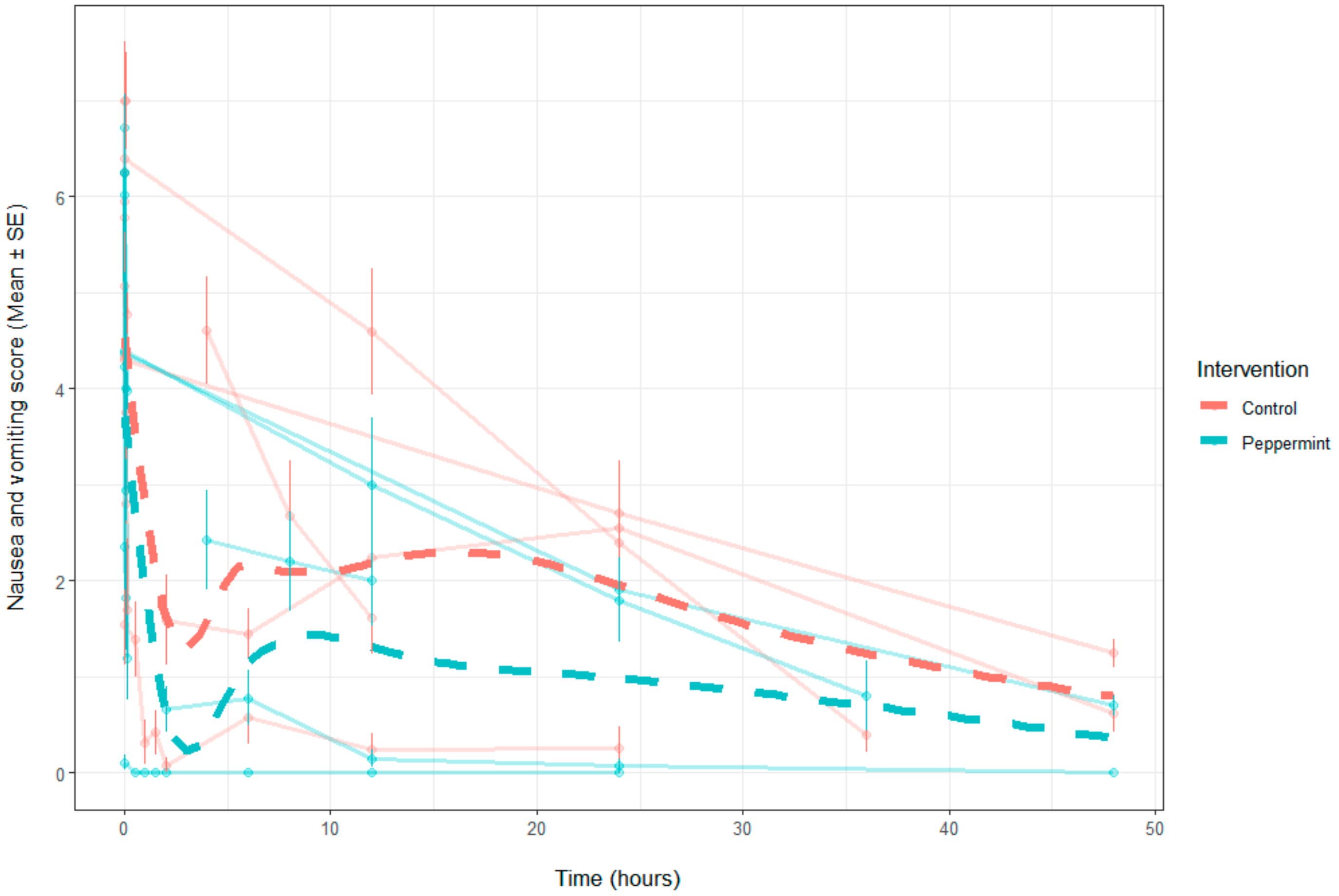

Figure S2 Moving averages of peppermint oil intervention versus control for PONV scores over time (hours)

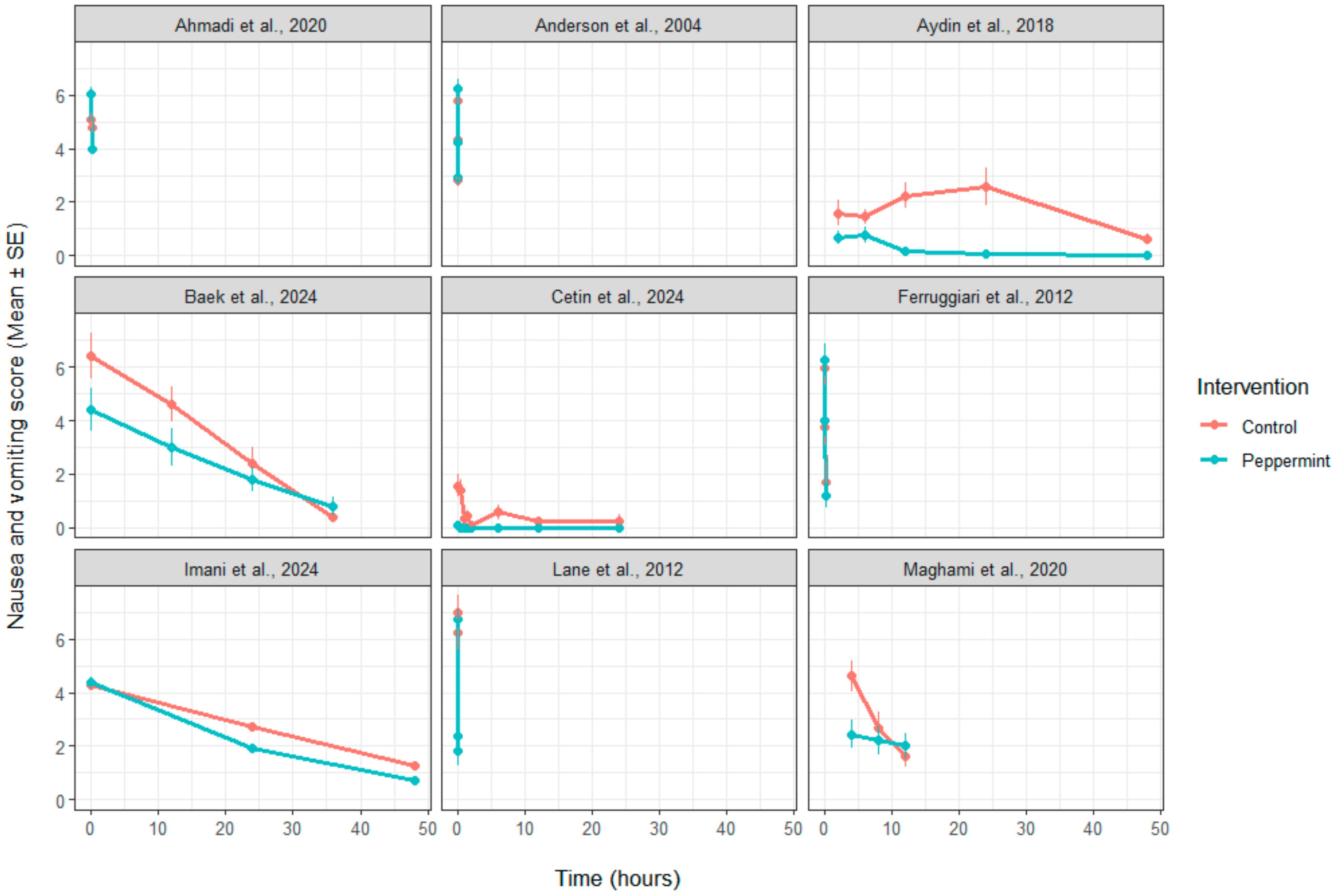

Figure S3 Individual study results comparing peppermint oil intervention versus control for PONV scores over time (hours) [28,33,51–57]

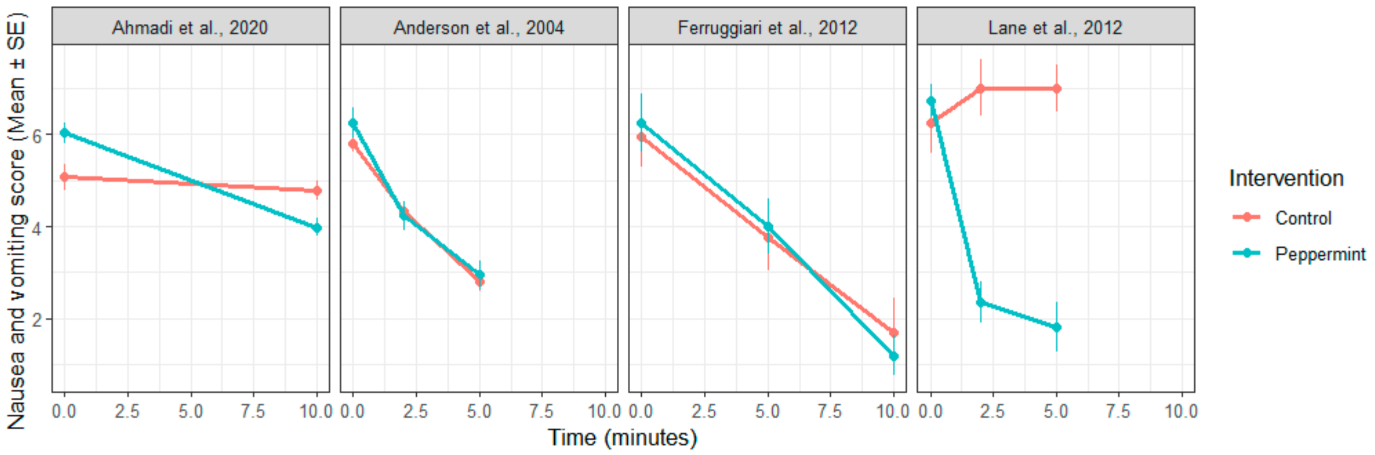

Figure S4 Individual study results comparing peppermint oil intervention versus control for PONV scores over time (minutes) [28,51,55,57]

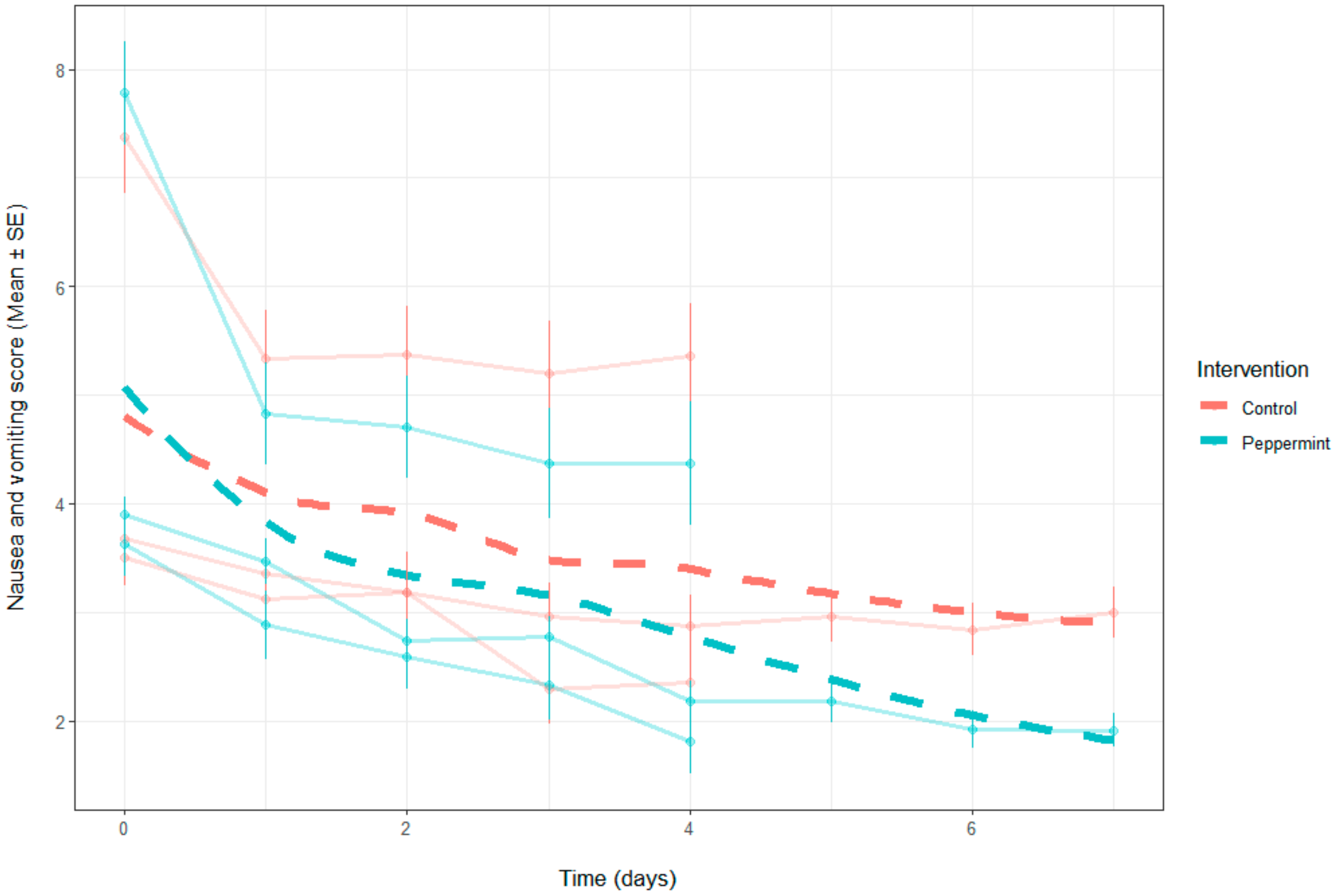

Figure S5 Moving averages of peppermint oil intervention versus control for NVP scores over time (days)

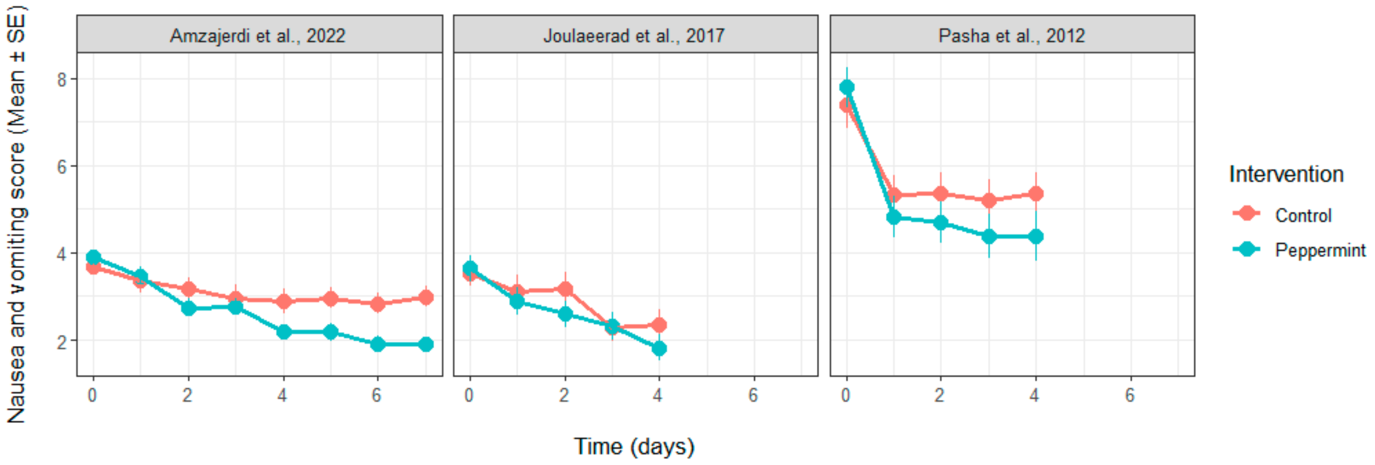

Figure S6 Individual study results comparing peppermint oil intervention versus control for NVP scores over time (days) [29,32,63]

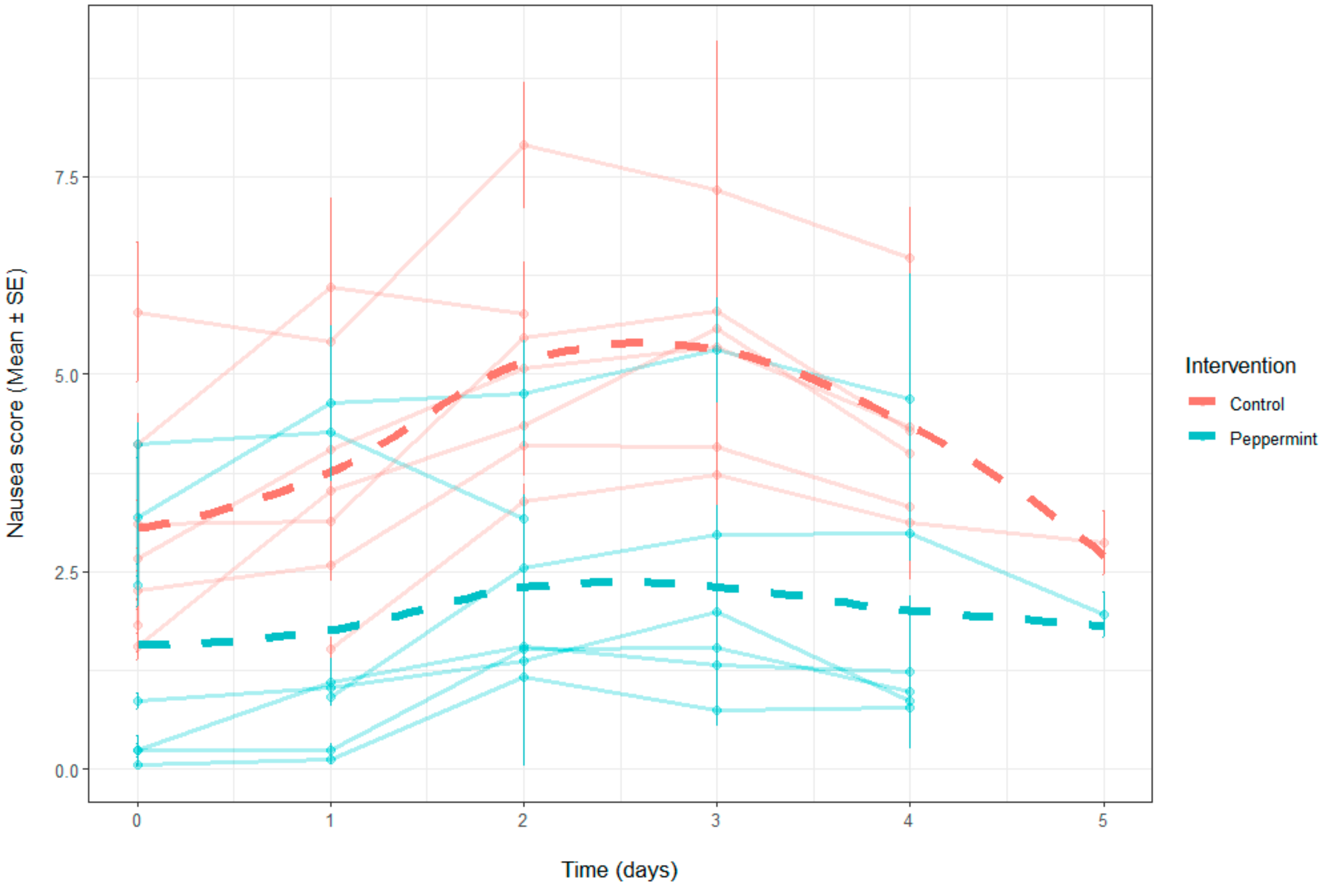

Figure S7 Moving averages of peppermint oil intervention versus control for CINV scores over time (days)

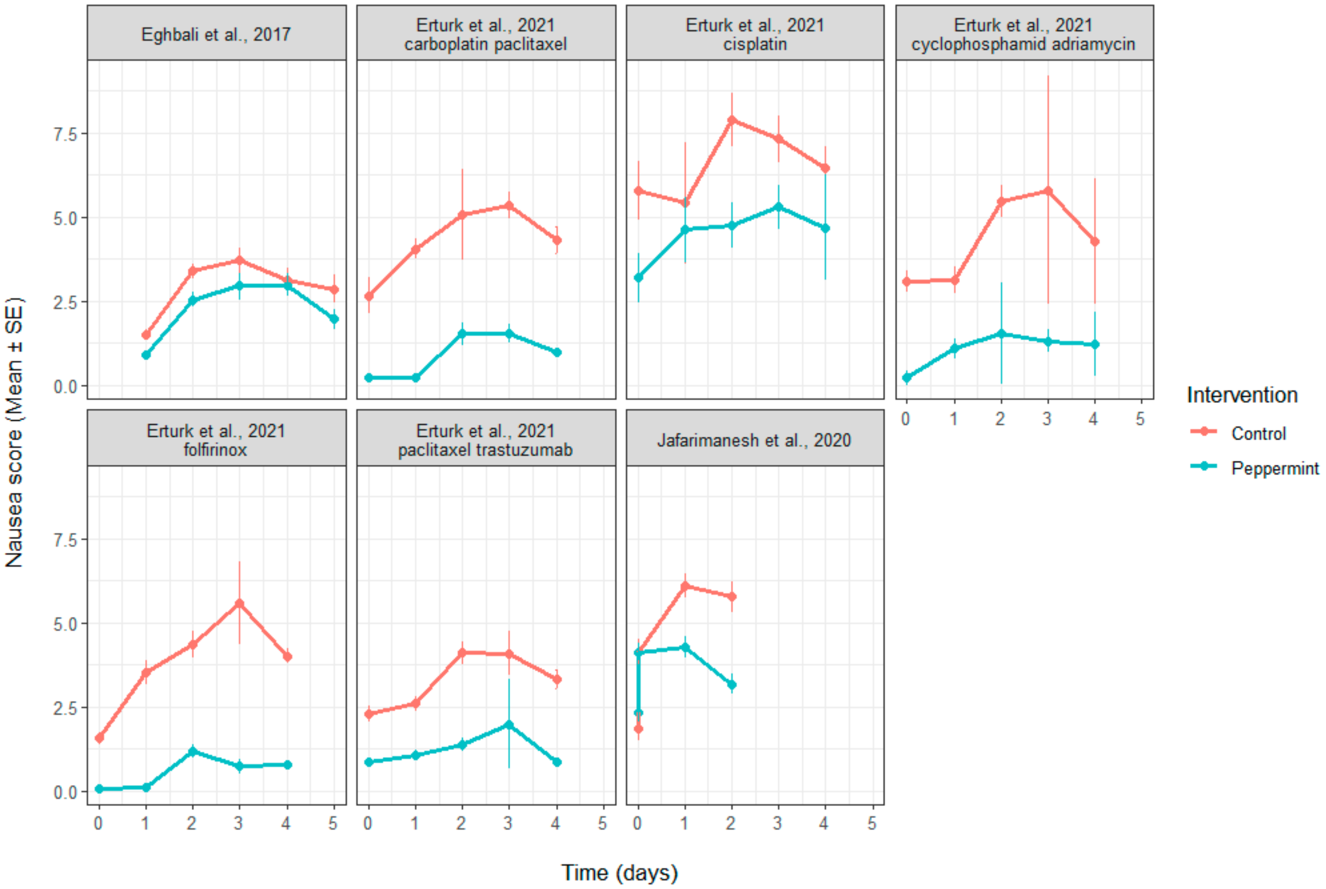

Figure S8 Individual study results comparing peppermint oil intervention versus control for CINV scores over time (days) [30,31,60]

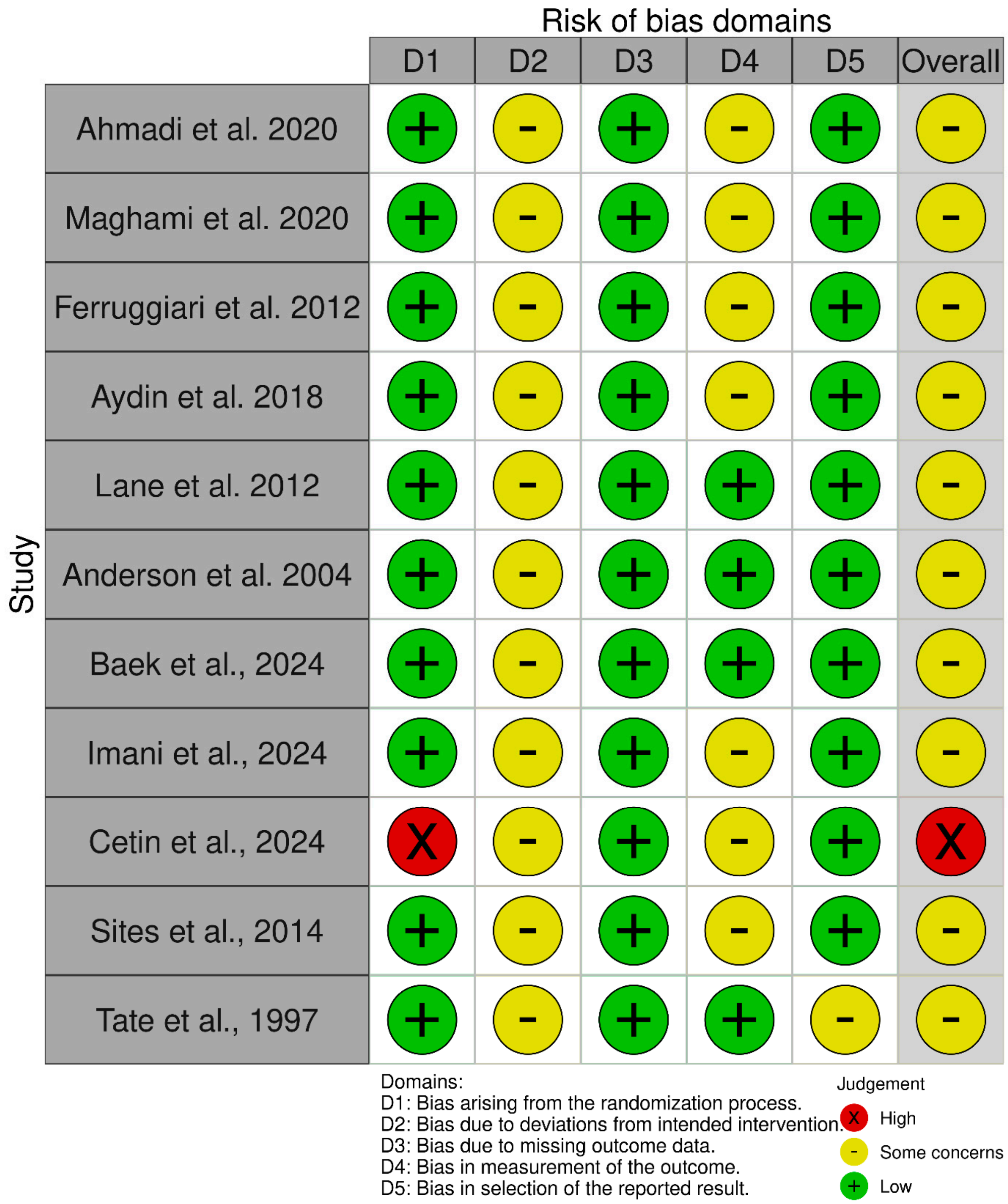

Figure S9 Risk of bias assessment of the included postoperative studies using the revised Cochrane risk-of-bias tool (RoB2) [28,33,51–59].

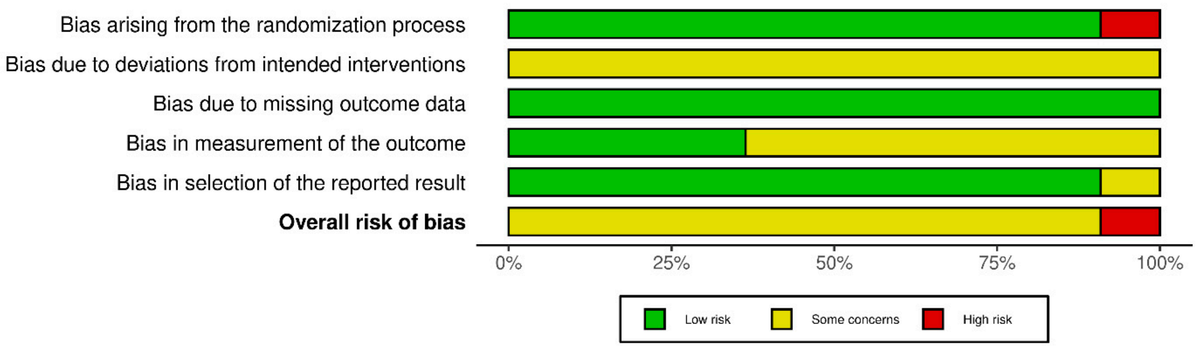

Figure S10 Risk of bias assessment of the included postoperative studies using the revised Cochrane risk-of-bias tool (RoB2) shown in percentage terms

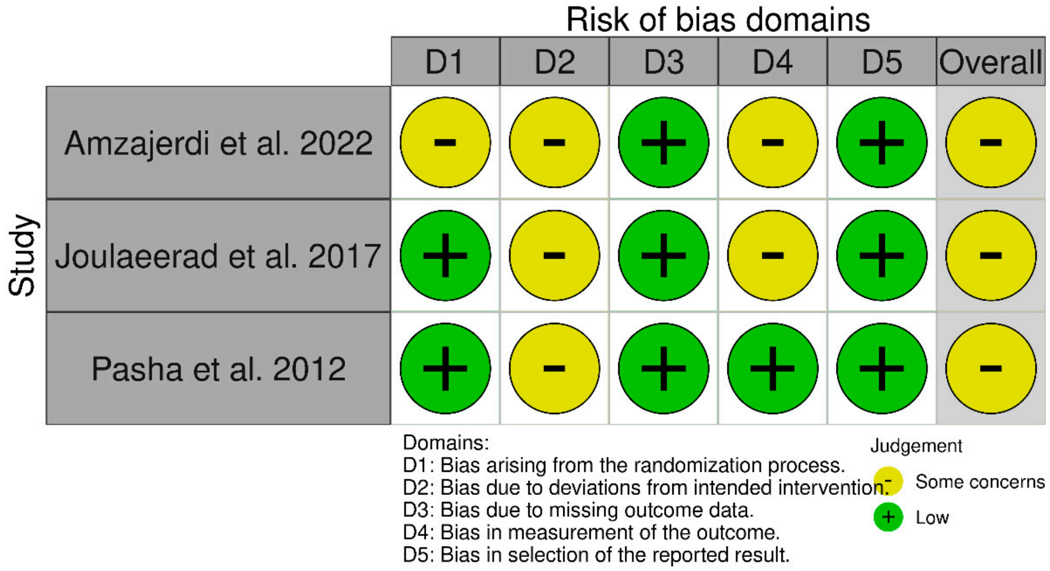

Figure S11 Risk of bias assessment of the included pregnancy studies using the revised Cochrane risk-of-bias tool (RoB2) [29,32,63]

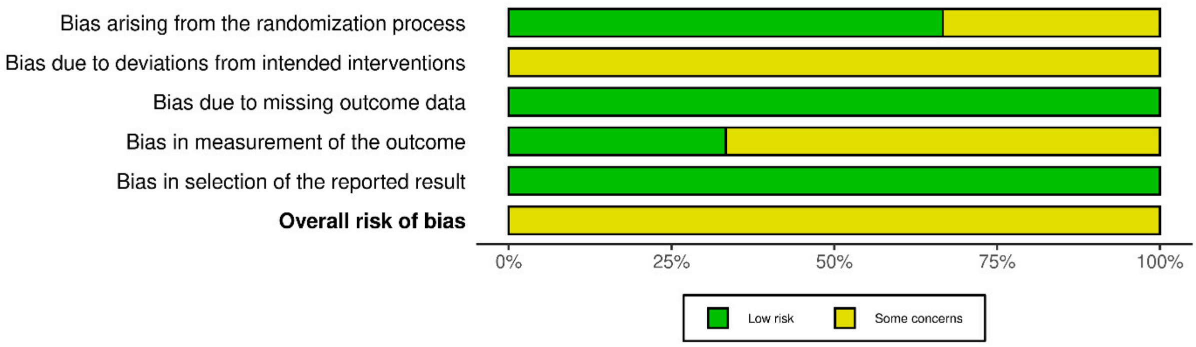

Figure S12 Risk of bias assessment of the included pregnancy studies using the revised Cochrane risk-of-bias tool (RoB2) shown in percentage terms

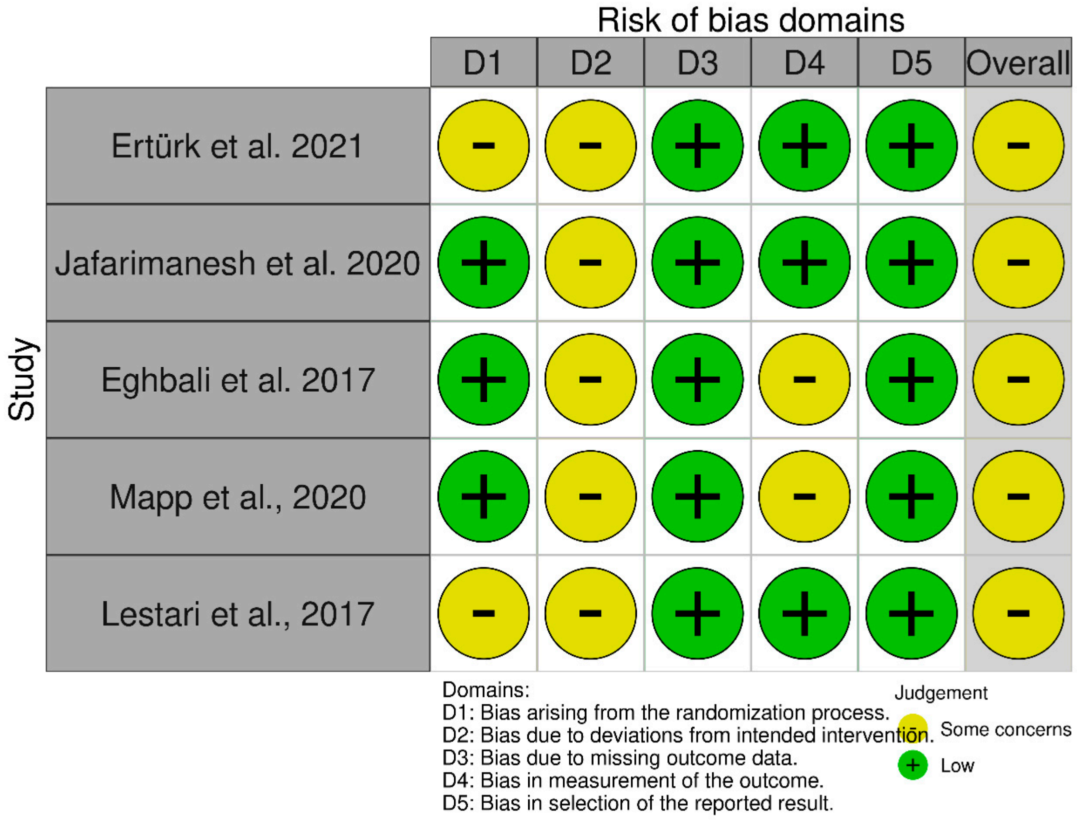

Figure S13 Risk of bias assessment of the included chemotherapy studies using the revised Cochrane risk-of-bias tool (RoB2) [30,31,60–62]

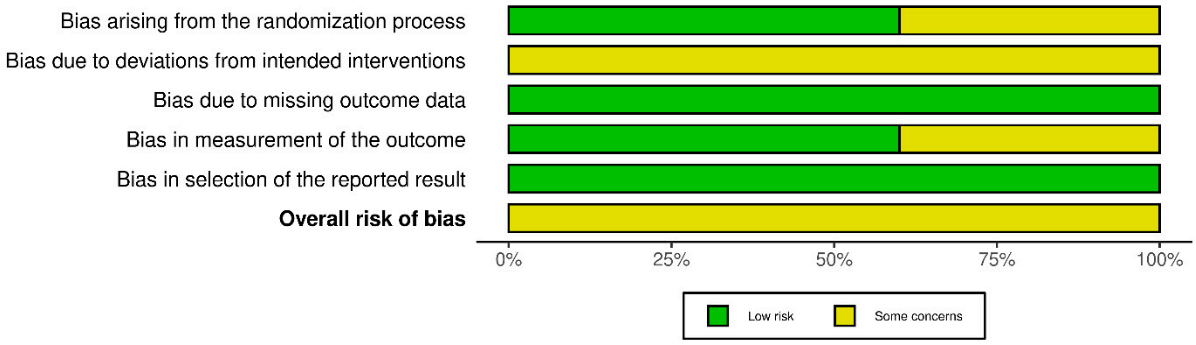

Figure S14 Risk of bias assessment of the included chemotherapy studies using the revised Cochrane risk-of-bias tool (RoB2) shown in percentage terms

McGuinness, LA, Higgins, JPT. Risk-of-bias VISualization (robvis): An R package and Shiny web app for visualizing risk-of-bias assessments. Res Syn Meth. 2020; 1- 7. https://doi.org/10.1002/jrsm.1411

Table S6 PRISMA 2020 Checklist

Section and Topic	Item #	Checklist item	Location where item is reported
**TITLE**			
Title	1	Identify the report as a systematic review.	row 1
**ABSTRACT**			
Abstract	2	See the PRISMA 2020 for Abstracts checklist.	row 21
**INTRODUCTION**			
Rationale	3	Describe the rationale for the review in the context of existing knowledge.	1
Objectives	4	Provide an explicit statement of the objective(s) or question(s) the review addresses.	1
**METHODS**			
Eligibility criteria	5	Specify the inclusion and exclusion criteria for the review and how studies were grouped for the syntheses.	2.1
Information sources	6	Specify all databases, registers, websites, organisations, reference lists and other sources searched or consulted to identify studies. Specify the date when each source was last searched or consulted.	2.2
Search strategy	7	Present the full search strategies for all databases, registers and websites, including any filters and limits used.	2.3
Selection process	8	Specify the methods used to decide whether a study met the inclusion criteria of the review, including how many reviewers screened each record and each report retrieved, whether they worked independently, and if applicable, details of automation tools used in the process.	2.4
Data collection process	9	Specify the methods used to collect data from reports, including how many reviewers collected data from each report, whether they worked independently, any processes for obtaining or confirming data from study investigators, and if applicable, details of automation tools used in the process.	2.5
Data items	10a	List and define all outcomes for which data were sought. Specify whether all results that were compatible with each outcome domain in each study were sought (e.g. for all measures, time points, analyses), and if not, the methods used to decide which results to collect.	2.6
	10b	List and define all other variables for which data were sought (e.g. participant and intervention characteristics, funding sources). Describe any assumptions made about any missing or unclear information.	2.6
Study risk of bias assessment	11	Specify the methods used to assess risk of bias in the included studies, including details of the tool(s) used, how many reviewers assessed each study and whether they worked independently, and if applicable, details of automation tools used in the process.	2.7
Effect measures	12	Specify for each outcome the effect measure(s) (e.g. risk ratio, mean difference) used in the synthesis or presentation of results.	2.9
Synthesis methods	13a	Describe the processes used to decide which studies were eligible for each synthesis (e.g. tabulating the study intervention characteristics and comparing against the planned groups for each synthesis (item #5)).	N/A
	13b	Describe any methods required to prepare the data for presentation or synthesis, such as handling of missing summary statistics, or data conversions.	2.9
	13c	Describe any methods used to tabulate or visually display results of individual studies and syntheses.	2.9
	13d	Describe any methods used to synthesize results and provide a rationale for the choice(s). If meta-analysis was performed, describe the model(s), method(s) to identify the presence and extent of statistical heterogeneity, and software package(s) used.	2.10
	13e	Describe any methods used to explore possible causes of heterogeneity among study results (e.g. subgroup analysis, meta-regression).	N/A
	13f	Describe any sensitivity analyses conducted to assess robustness of the synthesized results.	N/A
Reporting bias assessment	14	Describe any methods used to assess risk of bias due to missing results in a synthesis (arising from reporting biases).	N/A
Certainty assessment	15	Describe any methods used to assess certainty (or confidence) in the body of evidence for an outcome.	2.8

Section and Topic	Item #	Checklist item	Location where item is reported
**RESULTS**			
Study selection	16a	Describe the results of the search and selection process, from the number of records identified in the search to the number of studies included in the review, ideally using a flow diagram.	3.1
	16b	Cite studies that might appear to meet the inclusion criteria, but which were excluded, and explain why they were excluded.	NA
Study characteristics	17	Cite each included study and present its characteristics.	Table 1
Risk of bias in studies	18	Present assessments of risk of bias for each included study.	Figure S9-S14
Results of individual studies	19	For all outcomes, present, for each study: (a) summary statistics for each group (where appropriate) and (b) an effect estimate and its precision (e.g. confidence/credible interval), ideally using structured tables or plots.	3.3
Results of syntheses	20a	For each synthesis, briefly summarise the characteristics and risk of bias among contributing studies.	3.2.1
	20b	Present results of all statistical syntheses conducted. If meta-analysis was done, present for each the summary estimate and its precision (e.g. confidence/credible interval) and measures of statistical heterogeneity. If comparing groups, describe the direction of the effect.	3.2.1
	20c	Present results of all investigations of possible causes of heterogeneity among study results.	3.2.1
	20d	Present results of all sensitivity analyses conducted to assess the robustness of the synthesized results.	N/A
Reporting biases	21	Present assessments of risk of bias due to missing results (arising from reporting biases) for each synthesis assessed.	Figure S9-S14
Certainty of evidence	22	Present assessments of certainty (or confidence) in the body of evidence for each outcome assessed.	Table 2
**DISCUSSION**			
Discussion	23a	Provide a general interpretation of the results in the context of other evidence.	4
	23b	Discuss any limitations of the evidence included in the review.	4.1
	23c	Discuss any limitations of the review processes used.	4.1
	23d	Discuss implications of the results for practice, policy, and future research.	4.2
**OTHER INFORMATION**			
Registration and protocol	24a	Provide registration information for the review, including register name and registration number, or state that the review was not registered.	2
	24b	Indicate where the review protocol can be accessed, or state that a protocol was not prepared.	2
	24c	Describe and explain any amendments to information provided at registration or in the protocol.	N/A
Support	25	Describe sources of financial or non-financial support for the review, and the role of the funders or sponsors in the review.	row 595
Competing interests	26	Declare any competing interests of review authors.	row 610
Availability of data, code and other materials	27	Report which of the following are publicly available and where they can be found: template data collection forms; data extracted from included studies; data used for all analyses; analytic code; any other materials used in the review.	row 601

From: Page MJ, McKenzie JE, Bossuyt PM, Boutron I, Hoffmann TC, Mulrow CD, et al. The PRISMA 2020 statement: an updated guideline for reporting systematic reviews. BMJ 2021;372:n71. doi: 10.1136/bmj.n71
